# Bilateral sternoclavicular joint tubercular cold abscess

**DOI:** 10.4103/1817-1737.58966

**Published:** 2010

**Authors:** K. K. Pandita, Rajesh Sharma, Sandeep Dogra, Sarla Pandita

**Affiliations:** *Department of Medicine, Acharya Shri Chander College of Medical Sciences, Sidhra, Jammu, J&K, India. E-mail: panditakk@yahoo.co.in*; 1 *Department of Radiodiagnosis, Acharya Shri Chander College of Medical Sciences, Sidhra, Jammu, J&K, India.*; 2 *Department of Microbiology; Acharya Shri Chander College of Medical Sciences, Sidhra, Jammu, J&K, India.*; 3 *Department of Radiologist at District Hospital Samba, Jammu, J&K, India.*

Sir,

Tuberculosis continues to ravage the populations of developing countries. It can involve virtually all organ systems of the body.[1,2] Skeletal tuberculosis makes up about 10% of the extrapulmonary cases, out of which weight-bearing joints are most commonly involved.[1] We report a rare case of bilateral sternoclavicular joint cold abscess, due to tuberculosis. To the best of our knowledge, only one case of sternoclavicular joint tuberculosis with bilateral involvement, but without cold abscess formation, has been described.[3]

**Figure 1:**
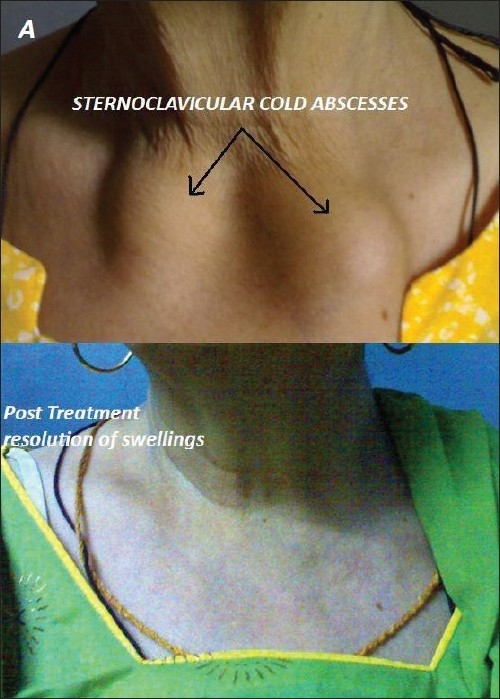
Swellings over sternoclavicular joints (upper) and resolution of swellings after antitubercular therapy (lower)

**Figure 2:**
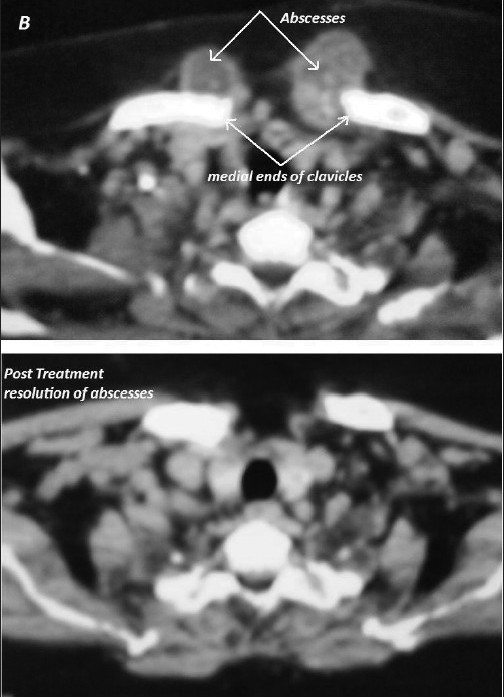
Computed tomography (CT) scan showing large fluid collections centred around bilateral sternoclavicular joints with erosions involving medial ends of clavicles and sternum (upper) and Follow up CT scan showing complete resolution of abscesses after antitubercular therapy (lower)

**Figure 3:**
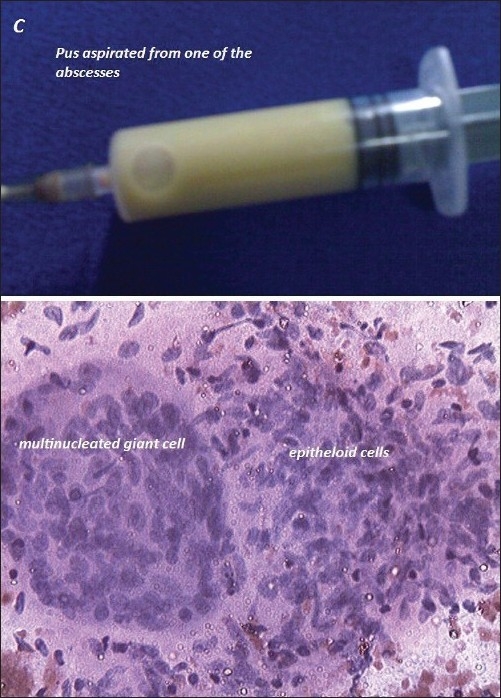
Pus from one of the swellings (upper) and fine needle aspiration of anterior mediastinal component showing epitheloid cell granulomas with multinucleated giant cells and necrosis (lower)

A 62-year-old woman presented with swellings over both sternoclavicular joints [Figure 1]. The swellings had grown gradually over a six-month period. She had a history of dry cough, weight loss, low-grade fever and night sweats. The temperature of the skin over the swellings was not raised. Computed tomography (CT) scan revealed large fluid collections in and around bilateral sternoclavicular joints with erosions involving medial ends of clavicles and sternum [Figure 2]. A heterogeneously enhancing soft tissue component was noted in the contiguous anterior mediastinum along with localized right-sided pleural effusion. Erythrocyte sedimentation rate was raised at 60 mm in the first hour. Needle aspiration of one of the swellings yielded pus [Figure 3]. No pyogenic organism was isolated from culture of pus. No acid-fast bacillus was seen on Ziehl-Nielsen staining of pus. Fine needle aspiration of anterior mediastinal component showed few epitheloid cell granulomas with multinucleated giant cells and necrosis (Panel C lower). Pleural fluid showed total leukocyte count of 840 cells/cu mm (90% lymphocytes and 10% polymorphs) with protein, glucose and lactate dehydrogenase content of 6.0 gm/deciliter, 70 milligrams/deciliter and 781 units/liter respectively. After receiving standard antitubercular therapy for one year, cold abscesses disappeared (Panel A lower) and she was asymptomatic. Follow-up CT scan showed complete resolution of abscesses (Panel B lower) and of pleural effusion with minimal residual soft tissue changes in anterior mediastinum.

Tubercular arthritis is typically monoarticular arthritis, resulting from hematogenous spread of primary infection.[1,4] It most commonly involves the spine followed by hip and knee joints. Other joints commonly involved include the sacroiliac, shoulder, elbow, ankle, carpal and tarsal joints.[1] The sternoclavicular joint is an extremely rare site for occurrence of tubercular arthritis.[3,5] It can be unilateral or bilateral.[2] This rarity of its occurrence can be attributed to the peculiar blood supply of this joint.[5] It most commonly presents as a cystic, globular, minimally tender, non-pulsatile swelling with no erythema or warmth over it.[2,3,5] Diagnosis is often delayed by many months due to the indolent nature of disease and absent or subtle constitutional symptoms.[4] CT scan of the joint shows destructive osseous changes.[3,4] In addition, it is helpful for defining the exact extent of disease.[4] Diagnosis becomes relatively easy, if acid-fast bacilli are isolated from aspirate or if aspirate reveals epitheloid cells or Langerhan's giant cells or if there is evidence of tuberculosis somewhere else (as in our patient, who had pleural effusion).[5] Otherwise definitive diagnosis may require surgical procedure for biopsy specimen. Empirical antitubercular therapy (ATT) may be started on the basis of clinical course and finding of cold abscess in the joint in areas where tuberculosis is endemic.[2] The conditions which enter into differential diagnosis of sternoclavicular cold abscess include low-grade pyogenic abscess, rheumatoid disease, myeloma and secondary deposits.[5] Early diagnosis is mandatory for good results, and with a worldwide resurgence of this disease, a high index of suspicion is mandatory. Standard ATT can cure the disease even when started late after development of cold abscess.
